# Crystal structure of ethyl 2-(2-fluoro­benzyl­idene)-5-(4-fluoro­phen­yl)-7-methyl-3-oxo-2,3-di­hydro-5*H*-1,3-thia­zolo[3,2-*a*]pyrimidine-6-carb­oxy­late

**DOI:** 10.1107/S1600536814025008

**Published:** 2014-11-19

**Authors:** M. S. Krishnamurthy, Noor Shahina Begum

**Affiliations:** a Department of Studies in Chemistry, Bangalore University, Bangalore 560 001, Karnataka, India

**Keywords:** crystal structure, fused pyrimidine derivative, fluoro-substituted compound, hydrogen bonding, C—H⋯π inter­actions, π–π inter­actions

## Abstract

In the title mol­ecule, C_23_H_18_F_2_N_2_O_3_S, the 4-fluoro-substituted and 2-fluoro-substituted benzene rings form dihedral angles of 88.16 (8) and 23.1 (1)°, respectively, with the thia­zole ring. The pyrimidine ring adopts a flattened sofa conformation with the *sp* ^3^-hydridized C atom forming the flap. In the crystal, pairs of weak C—H⋯O hydrogen bonds link mol­ecules related by twofold rotation axes, forming *R* ^2^_2_(10) rings, which are in turn linked by weak C—H⋯N inter­actions to form chains of rings along [010]. In addition, weak C—H⋯π(arene) inter­actions link the chains into layers parallel to (001) and π–π inter­actions with a centroid–centroid distance of 3.836 (10) Å connect these layers into a three-dimensional network.

## Related literature   

For the biological activity of fused pyrimidine derivatives, see: Alam *et al.* (2010*a* ▶,*b* ▶); Jotani *et al.* (2009 ▶). For the biological activity of fluoro-substituted compounds, see: Guru Row (1999 ▶); Yamazaki *et al.* (2009 ▶). For related structures, see: Krishnamurthy *et al.* (2014 ▶); Nagarajaiah & Begum (2011 ▶). For hydrogen-bond graph-set motifs motifs, see: Bernstein *et al.* (1995 ▶).
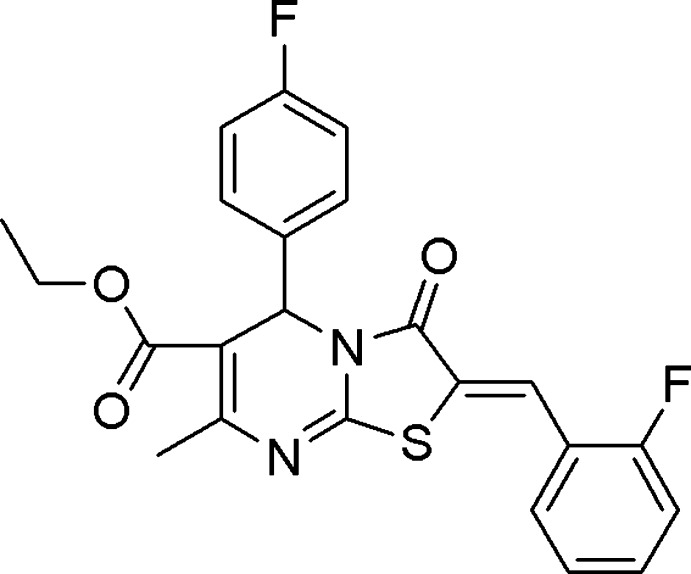


## Experimental   

### Crystal data  

C_23_H_18_F_2_N_2_O_3_S
*M* *_r_* = 440.45
Monoclinic, 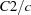

*a* = 24.746 (5) Å
*b* = 9.6879 (17) Å
*c* = 16.757 (3) Å
β = 92.022 (5)°
*V* = 4014.7 (13) Å^3^
*Z* = 8
Mo *K*α radiation
μ = 0.21 mm^−1^
*T* = 100 K
0.18 × 0.16 × 0.16 mm

### Data collection  

Bruker SMART APEX CCD diffractometer
Absorption correction: multi-scan (*SADABS*; Bruker, 1998 ▶) *T*_min_ = 0.963, *T*_max_ = 0.967
9957 measured reflections
3520 independent reflections
3169 reflections with *I* > 2σ(*I*)
*R*_int_ = 0.034

### Refinement  

*R*[*F* ^2^ > 2σ(*F* ^2^)] = 0.041
*wR*(*F* ^2^) = 0.112
*S* = 1.03
3520 reflections
282 parameters
H-atom parameters constrained
Δρ_max_ = 0.28 e Å^−3^
Δρ_min_ = −0.34 e Å^−3^

### 

Data collection: *SMART* (Bruker, 1998 ▶); cell refinement: *SAINT* (Bruker, 1998 ▶); data reduction: *SAINT*; program(s) used to solve structure: *SHELXS97* (Sheldrick, 2008 ▶); program(s) used to refine structure: *SHELXL97* (Sheldrick, 2008 ▶); molecular graphics: *ORTEP-3 for Windows* (Farrugia, 2012 ▶) and *DIAMOND* (Brandenburg & Berndt, 1999 ▶); software used to prepare material for publication: *WinGX* (Farrugia, 2012 ▶).

## Supplementary Material

Crystal structure: contains datablock(s) global, I. DOI: 10.1107/S1600536814025008/lh5738sup1.cif

Structure factors: contains datablock(s) I. DOI: 10.1107/S1600536814025008/lh5738Isup2.hkl

Supporting information file. DOI: 10.1107/S1600536814025008/lh5738Isup3.cml

**Fig. 1.**
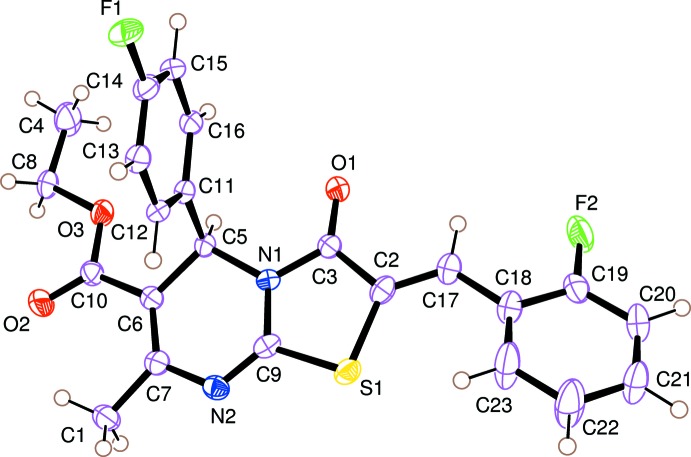
The molecular structure of the title compound drawn with 50% probability level ellipsoids. H atoms are presented as small spheres of arbitrary radius.

. DOI: 10.1107/S1600536814025008/lh5738fig1.tif
The mol­ecular structure of the title compound drawn with 50% probability level ellipsoids. H atoms are presented as small spheres of arbitrary radius.

**Fig. 2.**
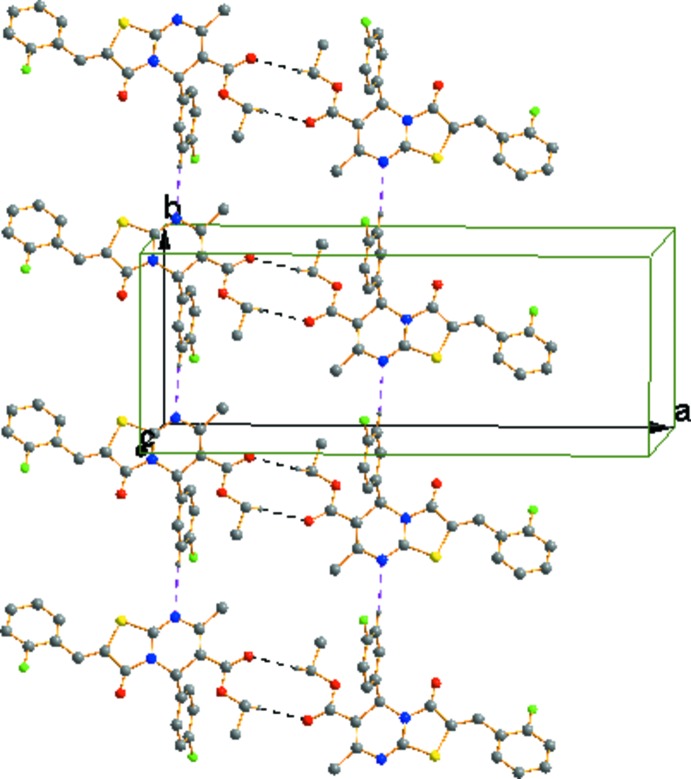
Part of the crystal structure with hydrogen bonds shown as dashed lines. For clarity H atoms not involved in hydrogen bonding are not shown.

. DOI: 10.1107/S1600536814025008/lh5738fig2.tif
Part of the crystal structure with hydrogen bonds shown as dashed lines. For clarity H atoms not involved in hydrogen bonding are not shown.

CCDC reference: 1034132

Additional supporting information:  crystallographic information; 3D view; checkCIF report

## Figures and Tables

**Table 1:** Hydrogen-bond geometry (, ) *Cg* is the centroid of the C11C16 ring.

*D*H*A*	*D*H	H*A*	*D* *A*	*D*H*A*
C8H8*A*O2^i^	0.99	2.48	3.119(2)	122
C15H15N2^ii^	0.95	2.57	3.488(2)	162
C8H8*B* *Cg* ^iii^	0.99	2.96	3.911(2)	162

Symmetry codes: (i) 
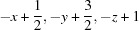
; (ii) 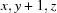; (iii) 
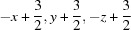
.

## supplementary crystallographic information

### S1. Comment

A heterocyclic nucleus imparts an important role in medicinal chemistry and serves as a key template for the development of various therapeutic agents. Among the various heterocyclic compounds, the synthetic studies of the fused pyrimidines that is specifically the thiazolo [3,2-a]pyrimidines have been reported extensively because of their structural diversity and association with a wide spectrum of biological activities such as antiviral, anticancer, anti-inflammatory and anti-hypertensive properties (Alam *et al.*, 2010*a*,*b*; Jotani *et al.*, 2009). The presence of a fluorine atom in the molecule can have profound and unexpected results on the biological activity of the compound (Guru Row, 1999; Yamazaki *et al.*, 2009). Herein, we report the crystal structure of the title compound (I).

The molecular structure of (I) is shown in Fig. 1. The 4-fluoro-substituted (C11–C16) and 2-fluoro-substituted (C18–C23) benzene rings form dihedral angles of 88.16 (8)° and 23.1 (1)°, respectively with the thiazole ring (C2/C3/N1/C9/S1). The pyrimidine ring adopts a flattened sofa conformation with atom C5 forming the flap. The carbonyl group of the exocyclic ester at C6 adopts a *cis* orientation with respect to C6═C7 double bond. The bond lengths and angles are in good agreement with those reported previously for related structures (Krishnamurthy *et al.*, 2014; Nagarajaiah & Begum *et al.*, 2011). In the crystal, pairs of weak C—H···O hydrogen bonds link molecules related by twofold rotation axes to form R^2^_2_(10) rings (Bernstein *et al.*, 1995), which are in turn linked by weak C—H···N interactions to form chains of rings (Fig. 2) along [010]. In addition, weak C—H···π(arene) interaction link chains into layers parallel to (001) and π···π interactions with a centroid–centroid distance of 3.836 (10)Å connect these layers into a three-dimensional network. The π···π interactions occur between symmetry related Cg–Cg(1-x, y, 3/2-z) rings, where Cg is the centroid of the C11–C16 ring.

### S2. Experimental

A mixture of 4-(4-fluoro-phenyl)-6-methyl-2-thioxo-1,2,3,4-tetrahydro- pyrimidine-5-carboxylic acid ethyl ester (10 mmol), chloroaceticacid (10 mmol), 2-fluorobenzaldehyde (10 mmol) and sodium acetate (1.5 g) was placed in a mixture of glacial acetic acid and acetic anhydride (25 ml; 1:1) and refluxed for 8—10 h until the TLC assay indicated that the reaction was complete. The reaction mixture was concentrated and the solid thus obtained was filtered and recrystallized from ethyl acetate to get the title compound (82% yield, mp 444 K). The compound was recrystallized by slow evaporation from dimethylformamide (DMF) solvent, yielding pale yellow single crystals suitable for X-ray diffraction studies.

### S3. Refinement

The H atoms were placed in calculated positions in the riding-model approximation with C—H = 0.95 – 1.00 Å and *U*_iso_(H) = 1.5*U*_eq_(C) for methyl H atoms and *U*_iso_(H) = 1.2*U*_eq_(C) for other hydrogen atoms.

### Crystal data

**Table:**

C_23_H_18_F_2_N_2_O_3_S	*F*(000) = 1824
*M**_r_* = 440.45	*D*_x_ = 1.457 Mg m^−^^3^
Monoclinic, *C*2/*c*	Mo *K*α radiation, λ = 0.71073 Å
Hall symbol: -C 2yc	Cell parameters from 3520 reflections
*a* = 24.746 (5) Å	θ = 1.7–25.0°
*b* = 9.6879 (17) Å	µ = 0.21 mm^−^^1^
*c* = 16.757 (3) Å	*T* = 100 K
β = 92.022 (5)°	Block, yellow
*V* = 4014.7 (13) Å^3^	0.18 × 0.16 × 0.16 mm
*Z* = 8	

### Data collection

**Table:**

Bruker SMART APEX CCD diffractometer	3520 independent reflections
Radiation source: fine-focus sealed tube	3169 reflections with *I* > 2σ(*I*)
Graphite monochromator	*R*_int_ = 0.034
ω scans	θ_max_ = 25.0°, θ_min_ = 1.7°
Absorption correction: multi-scan (*SADABS*; Bruker, 1998)	*h* = −29→29
*T*_min_ = 0.963, *T*_max_ = 0.967	*k* = −11→11
9957 measured reflections	*l* = −16→19

### Refinement

**Table:**

Refinement on *F*^2^	Primary atom site location: structure-invariant direct methods
Least-squares matrix: full	Secondary atom site location: difference Fourier map
*R*[*F*^2^ > 2σ(*F*^2^)] = 0.041	Hydrogen site location: inferred from neighbouring sites
*wR*(*F*^2^) = 0.112	H-atom parameters constrained
*S* = 1.03	*w* = 1/[σ^2^(*F*_o_^2^) + (0.0629*P*)^2^ + 4.1702*P*] where *P* = (*F*_o_^2^ + 2*F*_c_^2^)/3
3520 reflections	(Δ/σ)_max_ = 0.001
282 parameters	Δρ_max_ = 0.28 e Å^−^^3^
0 restraints	Δρ_min_ = −0.34 e Å^−^^3^

### Special details

**Table:**

Geometry. All s.u.'s (except the s.u. in the dihedral angle between two l.s. planes) are estimated using the full covariance matrix. The cell s.u.'s are taken into account individually in the estimation of s.u.'s in distances, angles and torsion angles; correlations between s.u.'s in cell parameters are only used when they are defined by crystal symmetry. An approximate (isotropic) treatment of cell s.u.'s is used for estimating s.u.'s involving l.s. planes.
Refinement. Refinement of *F*^2^ against ALL reflections. The weighted *R*-factor *wR* and goodness of fit *S* are based on *F*^2^, conventional *R*-factors *R* are based on *F*, with *F* set to zero for negative *F*^2^. The threshold expression of *F*^2^ > 2σ(*F*^2^) is used only for calculating *R*-factors(gt) *etc*. and is not relevant to the choice of reflections for refinement. *R*-factors based on *F*^2^ are statistically about twice as large as those based on *F*, and *R*- factors based on ALL data will be even larger.

### Fractional atomic coordinates and isotropic or equivalent isotropic displacement parameters (Å^2^)

**Table:**

	*x*	*y*	*z*	*U*_iso_*/*U*_eq_	
S1	0.565115 (17)	0.43828 (4)	0.65490 (3)	0.02504 (15)	
F1	0.43031 (4)	1.13600 (11)	0.77686 (7)	0.0351 (3)	
O3	0.36126 (5)	0.76825 (13)	0.48959 (7)	0.0266 (3)	
O1	0.56122 (5)	0.78762 (12)	0.54420 (7)	0.0244 (3)	
N1	0.50281 (5)	0.61793 (14)	0.58253 (8)	0.0194 (3)	
N2	0.45904 (6)	0.41667 (14)	0.62854 (9)	0.0238 (3)	
F2	0.75173 (5)	0.66324 (14)	0.56697 (9)	0.0542 (4)	
C6	0.40531 (7)	0.59402 (17)	0.56217 (10)	0.0201 (4)	
O2	0.30987 (5)	0.59424 (14)	0.53122 (10)	0.0430 (4)	
C5	0.45256 (6)	0.69513 (16)	0.56176 (10)	0.0196 (4)	
H5	0.4553	0.7335	0.5067	0.023*	
C3	0.55343 (7)	0.67752 (17)	0.57664 (9)	0.0200 (4)	
C10	0.35362 (7)	0.64859 (18)	0.52739 (11)	0.0245 (4)	
C15	0.44806 (7)	1.05927 (17)	0.64716 (11)	0.0257 (4)	
H15	0.4531	1.1518	0.6302	0.031*	
C2	0.59521 (7)	0.58692 (18)	0.61561 (10)	0.0227 (4)	
C11	0.44590 (6)	0.81437 (17)	0.61988 (10)	0.0197 (4)	
C17	0.64788 (7)	0.62041 (19)	0.61580 (10)	0.0254 (4)	
H17	0.6564	0.7051	0.5906	0.031*	
C7	0.40961 (7)	0.46736 (17)	0.59525 (10)	0.0221 (4)	
C16	0.45294 (6)	0.94976 (17)	0.59459 (11)	0.0228 (4)	
H16	0.4612	0.9674	0.5406	0.027*	
C9	0.50089 (7)	0.49166 (17)	0.61977 (10)	0.0214 (4)	
C12	0.43361 (6)	0.78875 (17)	0.69901 (10)	0.0210 (4)	
H12	0.4287	0.6964	0.7165	0.025*	
C13	0.42838 (7)	0.89693 (18)	0.75269 (10)	0.0236 (4)	
H13	0.4201	0.8801	0.8068	0.028*	
C14	0.43566 (7)	1.02957 (18)	0.72492 (11)	0.0250 (4)	
C1	0.36551 (7)	0.36219 (19)	0.60085 (11)	0.0292 (4)	
H1A	0.3303	0.4078	0.5942	0.044*	
H1B	0.3681	0.3171	0.6532	0.044*	
H1C	0.3692	0.2929	0.5588	0.044*	
C18	0.69365 (7)	0.5428 (2)	0.64997 (11)	0.0285 (4)	
C8	0.31251 (7)	0.8348 (2)	0.45669 (12)	0.0315 (4)	
H8A	0.2865	0.8508	0.4993	0.038*	
H8B	0.2950	0.7755	0.4152	0.038*	
C19	0.74540 (7)	0.5658 (2)	0.62342 (11)	0.0299 (4)	
C20	0.79025 (8)	0.4941 (2)	0.65104 (12)	0.0368 (5)	
H20	0.8249	0.5135	0.6311	0.044*	
C21	0.78402 (8)	0.3936 (3)	0.70814 (13)	0.0425 (5)	
H21	0.8143	0.3413	0.7271	0.051*	
C4	0.32924 (9)	0.9693 (2)	0.42117 (15)	0.0472 (6)	
H4A	0.3483	1.0250	0.4621	0.071*	
H4B	0.2971	1.0192	0.4011	0.071*	
H4C	0.3533	0.9519	0.3771	0.071*	
C22	0.73389 (9)	0.3693 (3)	0.73753 (16)	0.0636 (8)	
H22	0.7296	0.3010	0.7775	0.076*	
C23	0.68945 (9)	0.4436 (3)	0.70917 (14)	0.0552 (7)	
H23	0.6551	0.4263	0.7308	0.066*	

### Atomic displacement parameters (Å^2^)

**Table:**

	*U*^11^	*U*^22^	*U*^33^	*U*^12^	*U*^13^	*U*^23^
S1	0.0253 (3)	0.0241 (3)	0.0257 (3)	0.00599 (16)	0.00118 (18)	0.00482 (17)
F1	0.0385 (6)	0.0243 (6)	0.0427 (7)	0.0029 (5)	0.0040 (5)	−0.0127 (5)
O3	0.0179 (6)	0.0298 (7)	0.0321 (7)	0.0029 (5)	0.0001 (5)	0.0065 (5)
O1	0.0208 (6)	0.0234 (6)	0.0292 (7)	0.0007 (5)	0.0034 (5)	0.0021 (5)
N1	0.0193 (7)	0.0177 (7)	0.0214 (7)	0.0029 (5)	0.0020 (6)	0.0010 (6)
N2	0.0266 (8)	0.0207 (7)	0.0243 (8)	0.0006 (6)	0.0026 (6)	0.0014 (6)
F2	0.0245 (6)	0.0584 (8)	0.0795 (10)	−0.0091 (6)	−0.0015 (6)	0.0307 (7)
C6	0.0198 (8)	0.0212 (8)	0.0195 (8)	−0.0013 (7)	0.0029 (6)	−0.0030 (7)
O2	0.0199 (7)	0.0314 (7)	0.0774 (11)	−0.0033 (6)	−0.0031 (7)	0.0116 (7)
C5	0.0171 (8)	0.0194 (8)	0.0221 (8)	0.0026 (6)	0.0005 (6)	0.0022 (7)
C3	0.0209 (8)	0.0214 (8)	0.0180 (8)	0.0017 (7)	0.0033 (6)	−0.0018 (7)
C10	0.0217 (9)	0.0222 (8)	0.0298 (9)	−0.0001 (7)	0.0032 (7)	−0.0030 (7)
C15	0.0207 (9)	0.0168 (8)	0.0399 (11)	0.0003 (6)	0.0035 (8)	0.0025 (7)
C2	0.0243 (9)	0.0264 (9)	0.0176 (8)	0.0055 (7)	0.0023 (7)	−0.0019 (7)
C11	0.0136 (7)	0.0204 (8)	0.0251 (9)	0.0011 (6)	0.0000 (6)	0.0001 (7)
C17	0.0239 (9)	0.0308 (9)	0.0217 (9)	0.0036 (7)	0.0014 (7)	−0.0013 (7)
C7	0.0233 (9)	0.0227 (8)	0.0206 (8)	−0.0002 (7)	0.0049 (7)	−0.0033 (7)
C16	0.0183 (8)	0.0228 (9)	0.0274 (9)	0.0012 (6)	0.0026 (7)	0.0030 (7)
C9	0.0257 (9)	0.0202 (8)	0.0186 (8)	0.0043 (7)	0.0026 (7)	−0.0011 (7)
C12	0.0181 (8)	0.0177 (8)	0.0271 (9)	0.0007 (6)	0.0005 (7)	0.0032 (7)
C13	0.0196 (8)	0.0265 (9)	0.0248 (9)	0.0028 (7)	0.0009 (7)	−0.0009 (7)
C14	0.0186 (8)	0.0215 (8)	0.0349 (10)	0.0028 (7)	0.0002 (7)	−0.0081 (7)
C1	0.0291 (10)	0.0265 (9)	0.0322 (10)	−0.0059 (8)	0.0049 (8)	0.0019 (8)
C18	0.0211 (9)	0.0402 (10)	0.0241 (9)	0.0053 (8)	−0.0003 (7)	−0.0038 (8)
C8	0.0185 (9)	0.0344 (10)	0.0412 (11)	0.0042 (7)	−0.0049 (8)	0.0055 (9)
C19	0.0240 (9)	0.0342 (10)	0.0312 (10)	−0.0031 (8)	−0.0018 (7)	0.0000 (8)
C20	0.0177 (9)	0.0523 (13)	0.0402 (11)	−0.0006 (8)	−0.0014 (8)	−0.0022 (10)
C21	0.0252 (10)	0.0627 (14)	0.0392 (12)	0.0123 (10)	−0.0038 (9)	0.0083 (11)
C4	0.0290 (11)	0.0514 (13)	0.0612 (15)	0.0061 (9)	0.0010 (10)	0.0276 (12)
C22	0.0288 (12)	0.102 (2)	0.0601 (16)	0.0125 (13)	0.0013 (11)	0.0483 (16)
C23	0.0227 (11)	0.094 (2)	0.0489 (14)	0.0123 (11)	0.0073 (9)	0.0370 (14)

### Geometric parameters (Å, º)

**Table:**

S1—C9	1.7532 (17)	C17—H17	0.9500
S1—C2	1.7596 (18)	C7—C1	1.498 (2)
F1—C14	1.359 (2)	C16—H16	0.9500
O3—C10	1.338 (2)	C12—C13	1.390 (2)
O3—C8	1.459 (2)	C12—H12	0.9500
O1—C3	1.216 (2)	C13—C14	1.381 (3)
N1—C9	1.375 (2)	C13—H13	0.9500
N1—C3	1.386 (2)	C1—H1A	0.9800
N1—C5	1.482 (2)	C1—H1B	0.9800
N2—C9	1.278 (2)	C1—H1C	0.9800
N2—C7	1.414 (2)	C18—C23	1.387 (3)
F2—C19	1.349 (2)	C18—C19	1.389 (3)
C6—C7	1.349 (2)	C8—C4	1.497 (3)
C6—C10	1.484 (2)	C8—H8A	0.9900
C6—C5	1.526 (2)	C8—H8B	0.9900
O2—C10	1.208 (2)	C19—C20	1.376 (3)
C5—C11	1.524 (2)	C20—C21	1.378 (3)
C5—H5	1.0000	C20—H20	0.9500
C3—C2	1.489 (2)	C21—C22	1.371 (3)
C15—C14	1.380 (3)	C21—H21	0.9500
C15—C16	1.387 (2)	C4—H4A	0.9800
C15—H15	0.9500	C4—H4B	0.9800
C2—C17	1.343 (3)	C4—H4C	0.9800
C11—C16	1.391 (2)	C22—C23	1.385 (3)
C11—C12	1.393 (2)	C22—H22	0.9500
C17—C18	1.460 (2)	C23—H23	0.9500
			
C9—S1—C2	91.35 (8)	C11—C12—H12	119.7
C10—O3—C8	115.66 (13)	C14—C13—C12	117.86 (16)
C9—N1—C3	116.66 (14)	C14—C13—H13	121.1
C9—N1—C5	120.90 (13)	C12—C13—H13	121.1
C3—N1—C5	121.74 (13)	F1—C14—C15	118.47 (16)
C9—N2—C7	116.77 (14)	F1—C14—C13	118.26 (16)
C7—C6—C10	122.66 (15)	C15—C14—C13	123.26 (16)
C7—C6—C5	122.46 (15)	C7—C1—H1A	109.5
C10—C6—C5	114.81 (14)	C7—C1—H1B	109.5
N1—C5—C11	109.96 (13)	H1A—C1—H1B	109.5
N1—C5—C6	108.16 (13)	C7—C1—H1C	109.5
C11—C5—C6	112.57 (13)	H1A—C1—H1C	109.5
N1—C5—H5	108.7	H1B—C1—H1C	109.5
C11—C5—H5	108.7	C23—C18—C19	115.70 (17)
C6—C5—H5	108.7	C23—C18—C17	124.10 (17)
O1—C3—N1	123.73 (15)	C19—C18—C17	120.21 (17)
O1—C3—C2	126.49 (15)	O3—C8—C4	107.36 (15)
N1—C3—C2	109.78 (14)	O3—C8—H8A	110.2
O2—C10—O3	123.02 (16)	C4—C8—H8A	110.2
O2—C10—C6	125.85 (17)	O3—C8—H8B	110.2
O3—C10—C6	111.13 (14)	C4—C8—H8B	110.2
C14—C15—C16	117.88 (16)	H8A—C8—H8B	108.5
C14—C15—H15	121.1	F2—C19—C20	118.49 (17)
C16—C15—H15	121.1	F2—C19—C18	117.92 (16)
C17—C2—C3	121.14 (16)	C20—C19—C18	123.58 (19)
C17—C2—S1	128.34 (14)	C19—C20—C21	118.82 (18)
C3—C2—S1	110.49 (12)	C19—C20—H20	120.6
C16—C11—C12	119.46 (16)	C21—C20—H20	120.6
C16—C11—C5	120.17 (15)	C22—C21—C20	119.71 (19)
C12—C11—C5	120.36 (14)	C22—C21—H21	120.1
C2—C17—C18	128.02 (18)	C20—C21—H21	120.1
C2—C17—H17	116.0	C8—C4—H4A	109.5
C18—C17—H17	116.0	C8—C4—H4B	109.5
C6—C7—N2	122.16 (15)	H4A—C4—H4B	109.5
C6—C7—C1	126.68 (16)	C8—C4—H4C	109.5
N2—C7—C1	111.15 (15)	H4A—C4—H4C	109.5
C15—C16—C11	120.87 (16)	H4B—C4—H4C	109.5
C15—C16—H16	119.6	C21—C22—C23	120.3 (2)
C11—C16—H16	119.6	C21—C22—H22	119.8
N2—C9—N1	126.81 (15)	C23—C22—H22	119.8
N2—C9—S1	121.49 (13)	C22—C23—C18	121.8 (2)
N1—C9—S1	111.69 (12)	C22—C23—H23	119.1
C13—C12—C11	120.67 (15)	C18—C23—H23	119.1
C13—C12—H12	119.7		
			
C9—N1—C5—C11	104.58 (16)	C9—N2—C7—C6	−5.9 (2)
C3—N1—C5—C11	−65.51 (19)	C9—N2—C7—C1	172.94 (15)
C9—N1—C5—C6	−18.7 (2)	C14—C15—C16—C11	−0.5 (2)
C3—N1—C5—C6	171.21 (14)	C12—C11—C16—C15	0.2 (2)
C7—C6—C5—N1	14.5 (2)	C5—C11—C16—C15	−178.72 (15)
C10—C6—C5—N1	−168.64 (13)	C7—N2—C9—N1	1.3 (2)
C7—C6—C5—C11	−107.15 (18)	C7—N2—C9—S1	−177.66 (12)
C10—C6—C5—C11	69.67 (18)	C3—N1—C9—N2	−176.87 (16)
C9—N1—C3—O1	178.82 (15)	C5—N1—C9—N2	12.6 (3)
C5—N1—C3—O1	−10.7 (2)	C3—N1—C9—S1	2.14 (18)
C9—N1—C3—C2	−1.3 (2)	C5—N1—C9—S1	−168.43 (11)
C5—N1—C3—C2	169.20 (13)	C2—S1—C9—N2	177.27 (15)
C8—O3—C10—O2	3.5 (3)	C2—S1—C9—N1	−1.80 (12)
C8—O3—C10—C6	−176.91 (14)	C16—C11—C12—C13	−0.1 (2)
C7—C6—C10—O2	7.4 (3)	C5—C11—C12—C13	178.90 (14)
C5—C6—C10—O2	−169.38 (18)	C11—C12—C13—C14	0.1 (2)
C7—C6—C10—O3	−172.13 (15)	C16—C15—C14—F1	−179.44 (14)
C5—C6—C10—O3	11.1 (2)	C16—C15—C14—C13	0.5 (3)
O1—C3—C2—C17	−2.0 (3)	C12—C13—C14—F1	179.61 (14)
N1—C3—C2—C17	178.08 (15)	C12—C13—C14—C15	−0.4 (3)
O1—C3—C2—S1	179.75 (14)	C2—C17—C18—C23	−21.2 (3)
N1—C3—C2—S1	−0.14 (17)	C2—C17—C18—C19	158.67 (19)
C9—S1—C2—C17	−176.98 (17)	C10—O3—C8—C4	177.01 (17)
C9—S1—C2—C3	1.09 (12)	C23—C18—C19—F2	−178.7 (2)
N1—C5—C11—C16	108.87 (17)	C17—C18—C19—F2	1.4 (3)
C6—C5—C11—C16	−130.46 (16)	C23—C18—C19—C20	1.8 (3)
N1—C5—C11—C12	−70.07 (18)	C17—C18—C19—C20	−178.07 (18)
C6—C5—C11—C12	50.6 (2)	F2—C19—C20—C21	−179.40 (19)
C3—C2—C17—C18	−179.02 (16)	C18—C19—C20—C21	0.0 (3)
S1—C2—C17—C18	−1.1 (3)	C19—C20—C21—C22	−1.5 (4)
C10—C6—C7—N2	−179.71 (15)	C20—C21—C22—C23	1.0 (4)
C5—C6—C7—N2	−3.1 (2)	C21—C22—C23—C18	1.0 (5)
C10—C6—C7—C1	1.6 (3)	C19—C18—C23—C22	−2.3 (4)
C5—C6—C7—C1	178.17 (16)	C17—C18—C23—C22	177.6 (2)

### Hydrogen-bond geometry (Å, º)

Cg is the centroid of the C11–C16 ring.

**Table:**

*D*—H···*A*	*D*—H	H···*A*	*D*···*A*	*D*—H···*A*
C8—H8*A*···O2^i^	0.99	2.48	3.119 (2)	122
C15—H15···N2^ii^	0.95	2.57	3.488 (2)	162
C8—H8*B*···*Cg*^iii^	0.99	2.96	3.911 (2)	162

Symmetry codes: (i) −*x*+1/2, −*y*+3/2, −*z*+1; (ii) *x*, *y*+1, *z*; (iii) −*x*+3/2, *y*+3/2, −*z*+3/2.
