# Supplementary figures and images for: Crystal structure of ethyl 2-(2-fluoro­benzyl­idene)-5-(4-fluoro­phen­yl)-7-methyl-3-oxo-2,3-di­hydro-5H-1,3-thia­zolo[3,2-a]pyrimidine-6-carb­oxy­late
Source: Acta Crystallogr Sect E Struct Rep Online. 2014 Nov 19;70(Pt 12):o1270–1. doi: 10.1107/S1600536814025008 (PMC4257371; doi:10.1107/S1600536814025008)

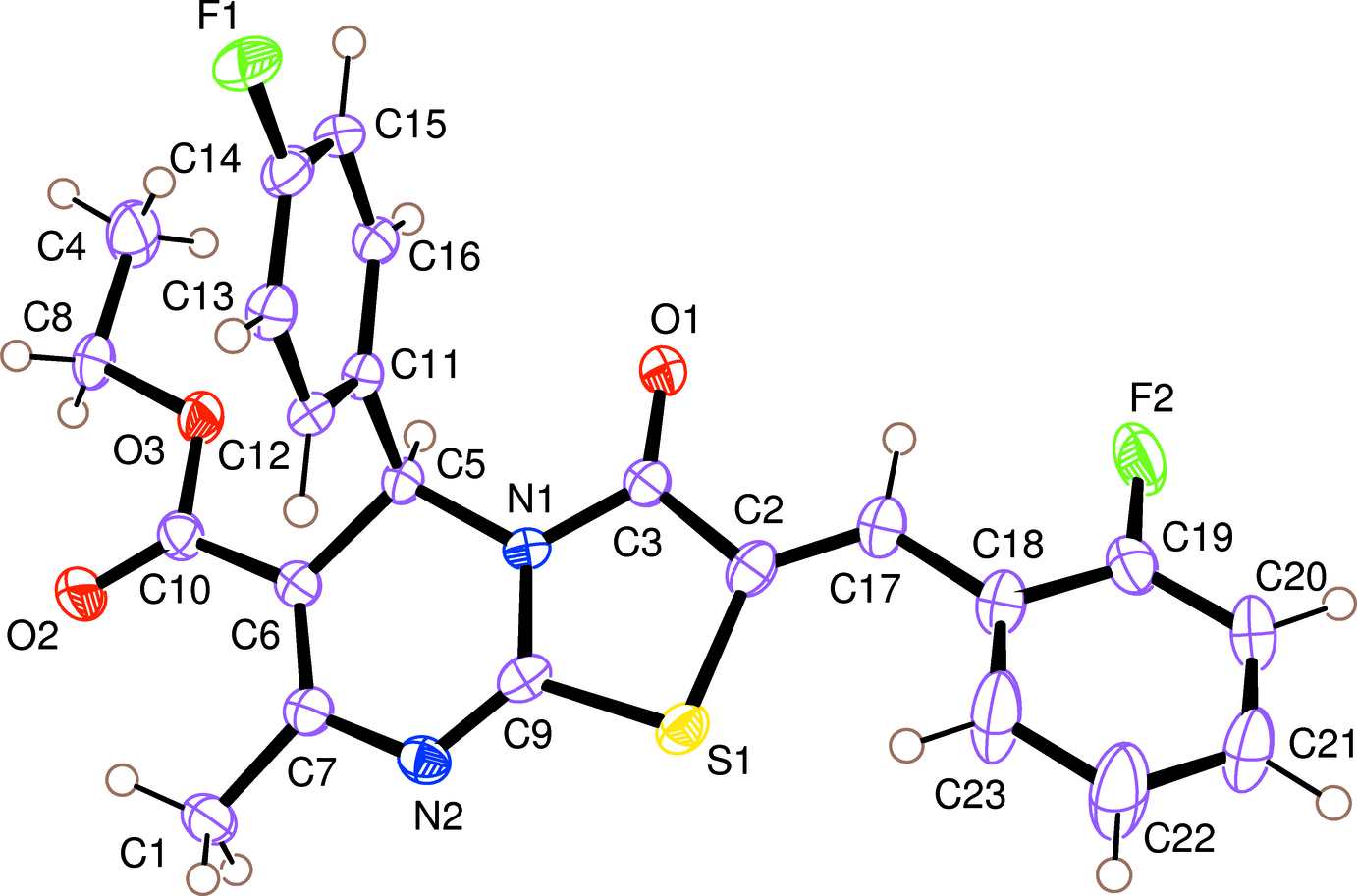

Supplement: Supplementary file 4 [file e-70-o1270-fig1.tif]

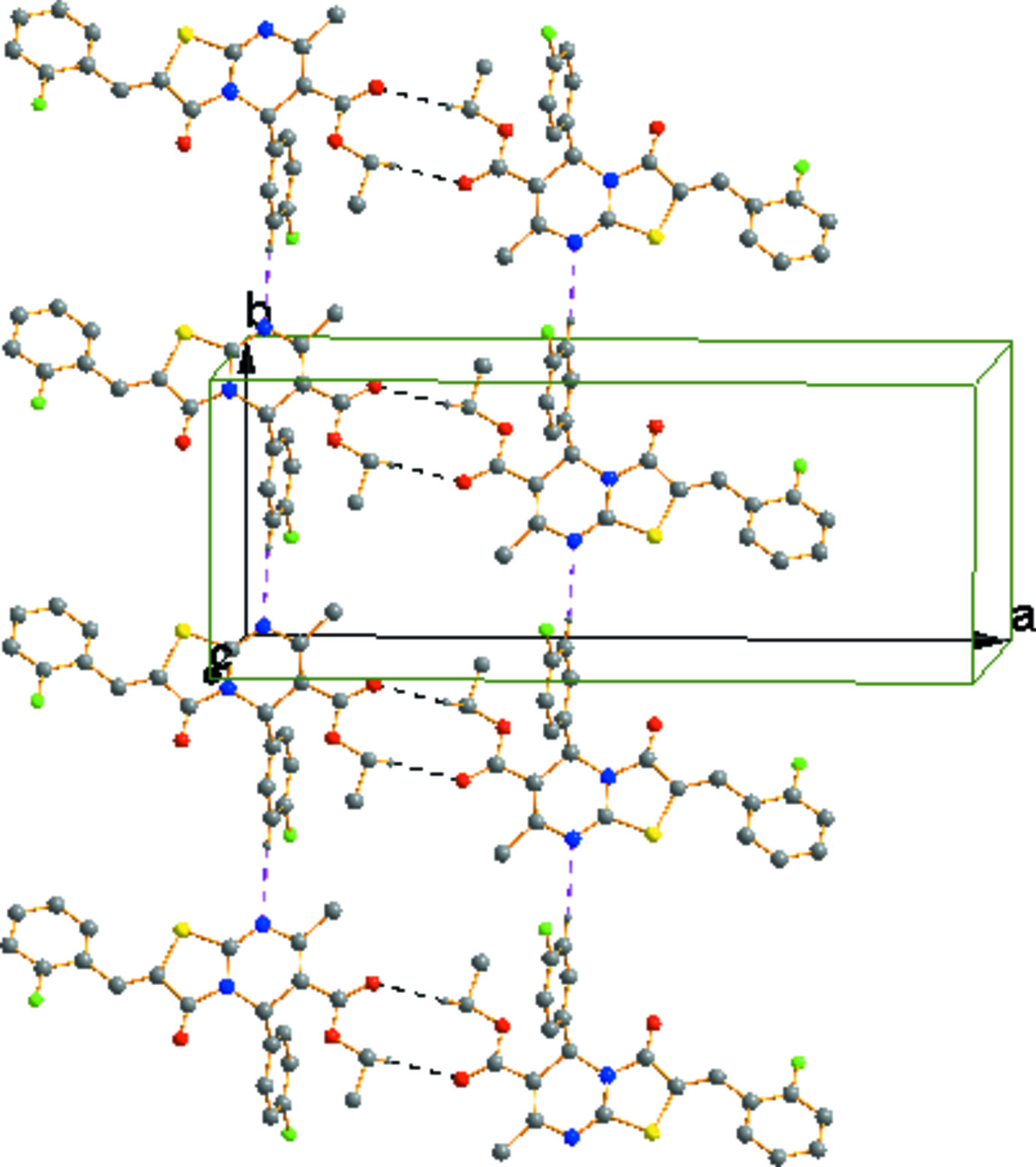

Supplement: Supplementary file 5 [file e-70-o1270-fig2.tif]
